# Development and pilot of an internationally standardized measure of cardiovascular risk management in European primary care

**DOI:** 10.1186/1472-6963-11-70

**Published:** 2011-04-07

**Authors:** Sabine Ludt, Stephen M Campbell, Jan van Lieshout, Richard Grol, Joachim Szecsenyi, Michel Wensing

**Affiliations:** 1 University of Heidelberg Hospital, Department of General Practice and Health Services Research, Voßstrasse 2, D-69115 Heidelberg, Germany; 2 National Primary Care Research and Development Centre, University of Manchester, Williamson Building, Manchester. M13 9PL, UK; 3 Radboud University Nijmegen Medical Centre, Scientific Institute for Quality of Healthcare, P.O. Box 9101, 6500 HB Nijmegen, the Netherlands

## Abstract

**Background:**

Primary care can play an important role in providing cardiovascular risk management in patients with established Cardiovascular Diseases (CVD), patients with a known high risk of developing CVD, and potentially for individuals with a low risk of developing CVD, but who have unhealthy lifestyles. To describe and compare cardiovascular risk management, internationally valid quality indicators and standardized measures are needed. As part of a large project in 9 European countries (EPA-Cardio), we have developed and tested a set of standardized measures, linked to previously developed quality indicators.

**Methods:**

A structured stepwise procedure was followed to develop measures. First, the research team allocated 106 validated quality indicators to one of the three target populations (established CVD, at high risk, at low risk) and to different data-collection methods (data abstraction from the medical records, a patient survey, an interview with lead practice GP/a practice survey). Secondly, we selected a number of other validated measures to enrich the assessment. A pilot study was performed to test the feasibility. Finally, we revised the measures based on the findings.

**Results:**

The EPA-Cardio measures consisted of abstraction forms from the medical-records data of established Coronary Heart Disease (CHD)-patients - and high-risk groups, a patient questionnaire for each of the 3 groups, an interview questionnaire for the lead GP and a questionnaire for practice teams. The measures were feasible and accepted by general practices from different countries.

**Conclusions:**

An internationally standardized measure of cardiovascular risk management, linked to validated quality indicators and tested for feasibility in general practice, is now available. Careful development and pilot testing of the measures are crucial in international studies of quality of healthcare.

## Background

Cardiovascular disease (CVD) is a major cause of premature death in Europe and also an important cause of morbidity, contributing substantially to escalating healthcare costs [1]. Cardiovascular risk management (CVRM) is targeted at three broad populations: those with established and diagnosed disease (e.g. angina, stroke), those at high risk of developing CVD due to known risk factors (e.g. hypertension, smoking), and individuals at low risk for developing CVD, but with unhealthy lifestyles. CVRM involves risk assessment and communicating to patients the risk of developing CVD [2,3], providing relevant guideline based treatments, such as counselling on lifestyle as well as cardio-protective medication and follow-up where appropriate [4,5]. Primary care has an important role in the delivery of CVRM and quality improvement programs have been developed to further strengthen this role [6].

Previous research has emphasised the importance of addressing global CVD risk assessment rather than focusing on individual risk factors such as smoking [5]. Various global CVD risk assessment and communication instruments for use in primary care have been developed across Europe [7-12], but there are differential and inconsistent use in routine clinical practice between and within countries [13-15]. The EUROASPIRE studies have advocated the need for more effective lifestyle management of patients at high risk and medication management of patients with coronary heart disease (CHD) [16]. These findings were derived from patients receiving treatment from medical specialists, whereas most patients received treatment in primary care, particularly in countries with a strong primary care system [17,18].

Quality indicators (QI) are used widely across Europe and internationally as part of quality improvement and pay-for-performance schemes [19-21], but research suggests that not all patients received the necessary evidence-based care that underpin these indicators [22,23].

We started a large project (EPA-Cardio) to assess CVRM in primary care across Europe. The participating countries were recruited from the TOPAS Europe Association, which is a collaboration between researchers of quality improvement in healthcare, founded in January 2005 http://www.epa-cardio.eu. In the first stage of the EPA-Cardio-project, we developed quality indicators for the prevention and management of cardiovascular disease in primary care [24]. Careful development and testing of measures linked to these quality indicators were deemed crucial for valid international comparisons. This paper describes the methods used to develop the set of measures, the results of a pilot in different countries of these measures, and the resulting final standardized EPA-Cardio instrument.

## Methods

The development of the data collection instruments and piloting took place between March 2007 and January 2008. A core group of researchers (SC, MW, JvL, and SL) developed the prototypes of the instruments, and discussed and refined it in meetings with the project partners. Pilot tests were done with GPs volunteering to participate.

### Development of the EPA-Cardio instrument

The EPA-Cardio instrument was developed by the project partners of the EPA-Cardio project, from 9 European countries, Austria, Belgium, Finland, France, Germany, Slovenia, Switzerland, the Netherlands and the UK. The measures were linked to the previously selected quality indicators (QI), using a modified Delphi Technique with expert panels in each participating country by a team, which was part of the first project [22,23]. The expert panels rated a core set of 44 indicators valid across 4 conceptual quality domains (lifestyle, clinical processes and outcomes, organisation and patient perspective). Furthermore, a broader set of additional 62 indicators had been rated "restricted valid" with a lower level of agreement. We decided to include these indicators in our study, because the core set mainly reflected the secondary prevention in patients with diabetes or established CVD, neglecting the primary prevention and patient perspective, which were represented in the wider set (broad set). (Table 1)

**Table 1:** Allocation of the quality indicators (QI) - Data collection methods and patient groups

Data collection method	Medical record audit				Patient survey				GP interview/ practice survey				overall number of QI
Quality of care domains*	LS	CP	O	PP	LS	CP	O	PP	LS	CP	O	PP	
**Patient groups**													
Patients with diabetes**	5 (4)	11 (3)	-	-	-	1 (0)	-	-	-	-	-	-	**17 (7)**
Patients with CVD	5 (4)	18 (9)	1 (1)	-	-	1 (0)	-	1 (0)	-	-	-	-	**26 (14)**
Patient at high risk for CVD	3 (0)	20 (7)	1 (1)	-	-	-	-	-	-	-	-	-	**24 (8)**
Primary prevention group	-	-	-	-	2 (0)	3 (0)	1 (0)	-	-	-	-	-	**6 (0)**
Practice organisation	-	-	-	-	-	-	-	-	-	18 (8)	15 (7)	-	**33 (15)**

**Overall number of QI**	**13** **(8)**	**49** **(19)**	**2** **(2)**	**0**	**2** **(0)**	**5** **(0)**	**1** **(0)**	**1** **(0)**	**0**	**18** **(8)**	**15** **(7)**	**0**	**106 (44)**

* LS = Lifestyle; CP = Clinical processes; O = Organisational aspects; PP = Patient's perspective

** The group of patients with diabetes had been excluded in the study

The numbers refer to the broad set of 106 quality indicators (QI) that were scored "restricted valid" with a lower level of agreement within all country panels.

The numbers in brackets refer to the core set of 44 quality indicators (QI), as part of the broad set, that were scored "valid" with a high level of agreement within all country panels.

**Figure 1:**
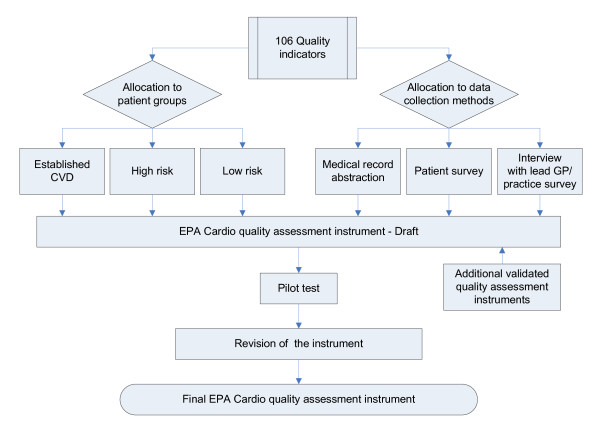
**The development of the EPA-Cardio instrument - a multi-stage process**.

The core group of researchers reviewed each of the 106 indicators and made 2 decisions per indicator (Figure 1): First, to which patient group was the indicator relevant; and second to the recommended data collection instruments. Indicators that were not applicable to a certain patient group, representing rather organisational aspects in general, were allocated to "general organisation" (table 1). As some quality of care-domains, such as patient perspective, and the "primary-prevention group" were under-represented in the quality indicator set, a number of additional measures (not linked to the quality indicators) were selected for subsequent research. The instruments were developed and agreed in a series of meetings between March and August 2007. All project partners discussed their disagreements, which led to several revisions of the instruments. The resulting EPA-Cardio instrument was developed in English and translated into relevant languages using forward and backward procedures [25].

### Patient populations (table 2)

**Table 2:** Inclusion- and exclusion criteria for different patient groups

Patient groups	1. Patients with CHD	2. Patients at high risk	3. Primary-prevention group
Inclusion criteria:	- Documented diagnosis	- Men ≥ 60 and	- Age: 18-45 years
	- ICD 10 code: I20-I25 or	- Smoker:	- Registered or regular visitors in practice
	- ICPC-2 code: K74-76	- ICD 10: F17 or	
		- ICPC-2: P17	
Exclusion criteria for all 3 groups:	- Diabetes		
	- terminal illness		
	- cognitive disorders (e.g. dementia)		
	- psychiatric diseases (e.g. schizophrenia)		
	- lack of language knowledge		
Exclusion criteria for patient groups 2 and 3		- established CVD (Angina, Myocardial infarction, stroke)	

Only the first two groups were included in the pilot study

#### Patients with established/diagnosed CHD

Some indicators related to CVD overall, and others in particularly to coronary heart disease (CHD). To facilitate data collection and homogenise our study group, we decided to focus on the patient group to whom all these indicators apply, i.e. patients with CHD. We excluded patients with diabetes because our primary focus was on patients with CVD.

#### Patients 'at high risk'

While we used widely accepted risk-calculation models to define high risk, for pragmatic reasons we defined 'high risk' for the EPA-Cardio pilot as a 'male smoker over 60 years of age. For these individuals (without other risk factors), the risk of fatal or nonfatal CVD events within 10 years was estimated at 14.4%, using a German electronic risk calculation instrument [10], meeting the definition-criteria of high risk. We expected to facilitate patient sampling by focusing on one risk factor other than age and gender.

#### Adults with unhealthy life styles

Only a few indicators related to this population. In EPA-Cardio, we focused on all individuals between 18 and 45 years. As risk and risk factors for CVD increases with age, we used this age limit with the intention of separating this group from patients at high risk of CVD.

### Patient samples

Data collection procedures differed between the participating countries, due to ethical and legal regulations, and the number of available practice team members. The research team provided guidance for patient sampling and data collection as part of the EPA-Cardio instrument.

#### Established CHD and 'at high risk' groups

In Germany, Slovenia, Switzerland, and the Netherlands, patient selection and data extraction of the medical record audit was conducted by a practice nurse (guided by the GP). Support by telephone and email was provided by a researcher in the relevant country. In Belgium, the GP took over this task, and, in the UK, a researcher visited the practice to conduct the searches and the audit in collaboration with practice staff.

Where there identified more than 30 patients in a practice, we used random numbers to select patients for inclusion. We included all identified patients where there were less than 30 patients. We used consecutive sampling if the practice conducted manual searches. All patients selected for the medical audit (established CHD and 'at high risk') per practice were included in the patient survey (maximum of 30 patients per group per practice). As in most countries (except the UK), informed consent was requested for auditing medical records, questionnaires with informed consent sheets were sent after selecting eligible patients, and data were abstracted only for those who had sent back written consents. We intended to include at least 15 patients of each group per practice.

#### Primary prevention group (aged 18-45)

It was planned to select the primary prevention group by electronic searches (considering age group and exclusion criteria) followed by random sampling of 40 individuals from the search list. Where an electronic search was not feasible, the practice approached regular visitors who met the inclusion criteria consecutively. As we aimed to include 15 individuals per practice, we planned to send 40 questionnaires per practice in cases of electronic selection, or to hand out 15 questionnaires to practice visitors ready to participate in the study.

### Measures

We allocated each indicator to the most appropriate instrument: data abstraction forms for the medical record for patients with CHD and patients at high risk respectively, patient questionnaires for all 3 groups as appropriate, an interview guide for the lead GP, and a questionnaire for practice members. The designing of the abstraction forms for the medical record followed that of Banks [26], in order to facilitate data encoding and analysis.

#### Questionnaire for patient survey

In all cases, selected patients were sent a questionnaire and letter explaining the aims of the study along with an informed consent sheet, a questionnaire (relevant to the appropriate group), and a self-addressed prepaid envelope to return the questionnaire to the research group and the written consent sheet to the practice team. The questionnaires included questions derived from the quality indicators, questions about sociodemographic issues and other conditions. Validated instruments to evaluate the quality of primary care from the patient perspective were also included for all 3 patient groups [27]. Other validated instruments were included to evaluate the quality of chronic care for patients with CHD [28-30] and instruments to assess lifestyle behaviour for patients at high risk and the primary prevention-group) [31-34].

#### Physician interview

A researcher conducted the interview with the lead GP or practice manager, either by telephone or face to face and recorded the answers in the interview questionnaire. The interview questionnaire contained questions representing indicators on organisational issues and risk assessment methods. In addition, practice engagement in cardiovascular quality improvement projects and public health projects on cardiovascular health were assessed, and also GP's views and activities on primary prevention.

#### Practice questionnaire

Practice members completed a practice questionnaire sent by the researchers. This questionnaire contained questions to identify the practice according to size, location, number and function of practice staff and number of listed or yearly attending patients. Other items were derived from the EPA practice management [35] and represented the EPA dimensions "information process and technology", "organisation of chronic care and prevention", and "quality improvement". These items were included to obtain an insight into the quality management activities of included practices considered to have an influence on study results.

### Pilot study

The aim of the pilot study was to assess the acceptability of the developed data collection instruments and feasibility of the methods, irrespective of the difference of nationally adapted methods. We conducted the pilot study at the end of 2007 in 2 practices in 6 of the participating countries Belgium, Germany, the Netherlands, Slovenia, Switzerland and the United Kingdom. Not all countries had the materials translated and ready in time for the pilot study. We focused only on patients with CHD and patients at high risk in the pilot, because most data-collection instruments and procedures were related to these groups and the primary-prevention group was easier to select. Researchers chose the practices out of national GP-networks, approaching them by phone or mail. In each of the participating countries, pilot practices received between 100€ and 400€ as an incentive to take part.

**Figure 2:**
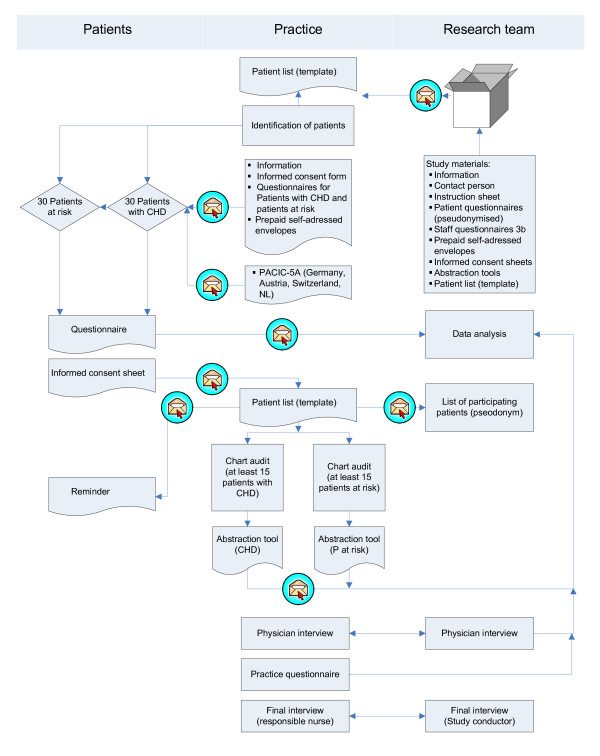
**EPA-Cardio pilot study - flow chart**.

All practices received a package with the study materials prior to the researcher visit (UK), or conducting the data collection in house (all other countries). This consisted of instruction sheets, the flow chart (Figure 2), questionnaires for patients, patient information, informed consent sheets and questionnaires for the practice teams.

Data collection procedures included patient selection, sending of patient questionnaires, data abstraction from the medical records, interview with the lead GP and completing of the practice questionnaire.

At the end of the pilot study, researchers interviewed the lead nurse by phone (face-to-face in the UK at the time of the visit), using an interview guide, and asked questions about the acceptability and feasibility of the instruments as well as the data-collection procedures. The pilot results were discussed at a meeting of all EPA-Cardio country co-ordinators, common standards were agreed, as also national adaptations to revise the instruments and procedures for subsequent use in the project. Ethical approval was sought as appropriate in the different participating countries.

## Results

The instrument was piloted in 12 European general practices, 2 general practices per country, in Belgium, Germany, the Netherlands, Slovenia, Switzerland and the UK. These practices selected a total of 268 patients with established CHD and 127 patients at high risk.

The practice teams rated study materials and instructions as easily understandable. Despite differences in data-collection procedures between the countries, all were capable of completing the assessment. It was possible to complete all procedures within 4-8 weeks. In the UK, an external reviewer completed the assessment within one working day per practice (without waiting for the replies from the patient's questionnaires).

### Data-collection procedures

#### Selection of patient groups, data abstraction

The group of patients with CHD was easier to select than the group of patients at high risk, because smoking status was not well documented in the medical records, and small practices could not select enough patients in this group. Therefore, it was possible to identify 30 patients with CHD in most practices, whereas the number of patients selected at high risk was between 8 and 30 (in larger practices with patient lists of >10000 patients).

#### Patient Questionnaires

The patients had some difficulties in completing the questionnaires, especially the PACIC-instrument, often asking nurses for explanations. Patient lists were used as check lists to follow the audit procedures, control the questionnaire responses, and send a reminder if necessary. Most of the countries sent reminders by mail. In Germany, the practice nurses reminded patients by telephone. Sending reminders was not permissible in the UK. The response rates were between 50% (UK) and 100% (Slovenia).

#### Interview with the lead GP

The researcher carried out the interview with the lead GP either in a face-to-face meeting (UK) or, in most countries, by telephone. The interview lasted 15-20 min.

#### Questionnaire for the practice team

Practice teams had no problems with completing the questionnaire for practice members. They needed 20-30 minutes to answer all questions.

#### Factors that contributed to the success or failure of data collection

There was a good level of practice co-operation with the research team of each country. Practices considered the instructions for patient selection and data-collection procedures as helpful, as well as the possibility to contact the research team if problems arose with study procedures. The practices reported that the selection of the eligible study patients from the medical records raised the major problem and consumed the most time of the audit. In most countries, nurses needed the help of the GP because they were not familiar with ICD or ICPC codes and searches run with the practice software.

### The final EPA-Cardio instrument

Finally, researchers of 9 European countries developed and agreed the final EPA-Cardio instrument (table 3). The instrument consisted of 4 types of paper-based instruments: abstraction forms to collect the data from the medical records, an interview questionnaire for the lead GP, patient questionnaires for 3 different patient groups, and a questionnaire for practice teams. The instrument included 74 quality indicators (37 core set and 37 broad set) that were previously agreed in the Delphi procedures. The main revisions in comparison to the pilot instruments were changes to the definition of the high-risk group to facilitate patient selection. We finally defined this group by risk calculation with recommended tools according to national guidelines, e.g. 10% fatal CVD-risk as calculated by the Dutch risk tables or by using a proxy measure: Patients with three out of the following four risk factors hypertension, hypercholesterolaemia, smoking and men over 60 years. The final EPA-Cardio instrument included a questionnaire for the primary-prevention group additionally. We also decided to apply to all the countries the PACIC-instrument that had been used in only 4 countries (Austria, Germany, the Netherlands and Switzerland) during the pilot. The changes to the instruments had to be translated again.

**Table 3:** The final EPA-Cardio instrument

Patients/Practice	Instruments/Data content	Number of items
**Data abstraction tools**		

Patient groups* 1 and 2	- Sex and age	- 2 items
	- Quality indicators (QI) on documentation of:	- 16 items for patient group 1 (18 QI)**
	- Patient's lifestyle (smoking, physical activity and BMI)	
	- lifestyle advice (smoking, physical activity and diet advice)	
	- levels of blood pressure, cholesterol and blood glucose	
		- 14 items for patient group 2 (12 QI)
Patient group 1	- QI on documentation of pharmaceutical treatment (statins, anti-platelet therapy and influenza vaccination)	- 3 items (3 QI)

**Interview guide**		

Lead GP	- QI on documentation an medical record	- 8 items (8 QI)
	- QI on CVD risk assessment	- 27 items (20 QI)
	- Practice engagement in CVD-quality improvement (programs) or	- 7 items (5 QI)
	- Practice engagement in CVD-related public health programs^+^	- 1 item
	- Views on primary prevention (lifestyle, lifestyle advice and support)^+^	- 11 items

**Patient questionnaires**		

All patient groups: 1,2,3	- Patient demographics and conditions^+^	- 11 items
	- Quality of primary care delivery (EUROPEP)^+^[27]	- 23 items
Patient group 1	- QI on lifestyle advice and patient perspective	- 2 items (2 QI)
	- Quality of chronic illness care (PACIC)^+^ [28;29]	- 26 items
Patient groups 1 and 2	- Quality of life / health state (EQ5D)^+^[51]	- 5 items and VAS
	- Medication/ Medication adherence (Morisky score) ^+^[30]	- 5 items
Patient groups 2 and 3	- Lifestyle: Rapid assessment of physical activity^+^ (RAPA) [32]	- 14 items
	- Lifestyle: Rapid Eating and Activity Assessment for participants - shortened version (REAP-S)^+^[33]	- 16 items
	- Lifestyle: Behavior Change Consortium: Smoking Assessment (Mid-Sized model - baseline measurements)^+^[34]	- 10 items
Patient group 3	- Views on primary prevention (lifestyle, lifestyle advice and support) ^+^	- 11 items (6 QI)

**Questionnaire for the practice team**^+^		

Practice team	- Information process and technology	- 11 items
	- Organisation of chronic care and prevention	- 20 items
	- Quality improvement activities	- 13 items
	- Practice size and location	- 5 items
	- Practice staff (number and function)	- 7 items

* Patient groups: 1 = Patients with CHD, 2 = Patients at high risk, 3 = Primary prevention group

** The information in brackets refers to the number of quality indicators (QI) included

^+ ^Supplementary instruments

VAS = Visual analogue scale

## Discussion

This study provided an internationally standardized measure of cardiovascular risk management in primary care, linked to previously selected quality indicators and pilot tested for feasibility.

### Strengths and limitations

The EPA-Cardio project was conducted by the same team that developed the EPA-practice management accreditation tool (**E**uropean **P**ractice **A**ssessment). Lessons learned in the process were applied to EPA-Cardio [35,36]. The basis of the EPA-Cardio instrument were quality indicators developed using a validated Delphi procedure. However, this methodology is did not cover all aspects of cardiovascular prevention and care as described and recommended in guidelines [20]. The assessment of important activities of primary care, such as communication, empathy, teamwork, consultation time, counselling and continuity of care, require other methodologies, such as in-depth interviews e.g. [37,38]. To complete the assessment of cardiovascular prevention and management, we supplemented the EPA-Cardio instrument with patient evaluation instruments. We therefore chose the EUROPEP-instrument because it is validated, used in many surveys and exists in the languages of all participants [27]. As patients' perspectives contribute considerably to the improvement in the quality of care [39], we also included the PACIC instrument to reflect the view of patients with established CHD [28]. This project aims to provide better cover of primary prevention, and we therefore included validated lifestyle-assessment instruments [31] and self-developed questions on attitudes towards primary prevention for individuals and GPs.

We included a convenience sample of countries out of the TOPAS Europe Association http://www.topaseurope.eu in the study for feasibility reasons and good experiences with collaboration in past projects [35,36]. This begs the question of whether EPA-Cardio be different if, for example, Greece and Denmark and Portugal had taken part in the study.

In the pilot, we used a small convenience sample of 2 practices in 6 of the 9 participating countries, as some countries were not able to have the study materials ready in time. The different methods used to select patient groups may lead to different populations (with limited generalisability) and different data-collection procedures, and therefore may restrict the comparability. The results of the subsequent observational study should therefore be interpreted carefully.

### Methods of data collection in general practices

The systematic collection of healthcare data across different countries in primary practice is difficult because of differences in levels of computerisation and different practice software within and between European countries [40]. The use of computers to record clinical data, and also the content and quality of electronic patient records, varies significantly [41,42]. In order not to exclude countries or practices with lower levels of computerisation, it is important to apply methodologies that prevent research only from being conducted selectively in technologically highly developed practices [22,43]. To make data collection feasible for practices with different technological levels in all countries, we developed paper-based abstraction tools. However, electronic data abstraction in general practice reduces the associated workload and is a pre-condition for the implementation of quality assessment in routine care [44].

### The challenge of cross-national assessment

The aim of the study was to develop standardized data collection procedures in all countries. However, it is also necessary to adapt to differences in national procedures to conduct cross-country collaborations. Perfect standardisation of data collection methods in cross-national studies is not possible due to differences in legislation and practice resources. For example, whether national data protection acts allow anyone not employed by the practice to look at patient medical records [45]. Another difficulty is the different standards of the ethics committees between, and even within, countries [46,47]; for example, in some countries practice staff are allowed to collect patient data for quality improvement purposes without the permission of patients (e.g. UK, the Netherlands), whereas in others (e.g. Germany, Belgium) the informed consent of the patient is required.

### The future of the EPA-Cardio instrument

We used the final EPA-Cardio instrument in the EPA-Cardio project within 9 participating countries between May 2008 and August 2009 [23]. The results of this large observational study may be used to compare countries with different quality improvement strategies, such as disease management programmes in Germany [48], indicator-based incentive contracts in the UK [49], or practice-based support with outreach visits in the Netherlands [50]. This may identify best practice examples and derive quality improvement recommendations for the prevention and management of cardiovascular diseases in Europe.

## Conclusions

An internationally standardized measure of CVRM in primary care is now available, linked to previously selected quality indicators. It has been pilot tested for feasibility. Careful development and pilot testing were deemed crucial for international comparisons. Detailed assessment, analysis and feedback can be the starting point for developing quality improvement activities and for deriving recommendations at a national and pan-European level.

## Competing interests

The authors declare that they have no competing interests.

## Authors' contributions

MW developed the overall outline of the EPA-Cardio project. SC co-ordinated the international selection of performance indicators, on which the EPA-Cardio audit instrument is based. SL co-ordinated the development and pilot testing of the measures and wrote the draft and the final version of this paper. JvL contributed to the development and selection of measurements. RG was the project leader of EPA-Cardio and JS was supervisor of the research reported in this paper. All authors critically assessed and approved this paper.

## Pre-publication history

The pre-publication history for this paper can be accessed here:

http://www.biomedcentral.com/1472-6963/11/70/prepub
